# Mitochondrial Damage Induced by T-2 Mycotoxin on Human Skin—Fibroblast Hs68 Cell Line

**DOI:** 10.3390/molecules28052408

**Published:** 2023-03-06

**Authors:** Edyta Janik-Karpinska, Michal Ceremuga, Marcin Niemcewicz, Ewelina Synowiec, Tomasz Sliwiński, Michal Bijak

**Affiliations:** 1 Biohazard Prevention Centre, Faculty of Biology and Environmental Protection, University of Lodz, Pomorska 141/143, 90-236 Lodz, Poland; 2 Military Institute of Armament Technology, Prymasa Stefana Wyszyńskiego 7, 05-220 Zielonka, Poland; 3 Laboratory of Medical Genetics, Faculty of Biology and Environmental Protection, University of Lodz, Pomorska 141/143, 90-236 Lodz, Poland

**Keywords:** T-2 toxin, mitochondria, mtDNA damage, Hs68 cell line, skin

## Abstract

T-2 toxin is produced by different *Fusarium* species and belongs to the group of type A trichothecene mycotoxins. T-2 toxin contaminates various grains, such as wheat, barley, maize, or rice, thus posing a risk to human and animal health. The toxin has toxicological effects on human and animal digestive, immune, nervous and reproductive systems. In addition, the most significant toxic effect can be observed on the skin. This in vitro study focused on T-2 toxicity on human skin fibroblast Hs68 cell line mitochondria. In the first step of this study, T-2 toxin’s effect on the cell mitochondrial membrane potential (MMP) was determined. The cells were exposed to T-2 toxin, which resulted in dose- and time-dependent changes and a decrease in MMP. The obtained results revealed that the changes of intracellular reactive oxygen species (ROS) in the Hs68 cells were not affected by T-2 toxin. A further mitochondrial genome analysis showed that T-2 toxin in a dose- and time-dependent manner decreased the number of mitochondrial DNA (mtDNA) copies in cells. In addition, T-2 toxin genotoxicity causing mtDNA damage was evaluated. It was found that incubation of Hs68 cells in the presence of T-2 toxin, in a dose- and time-dependent manner, increased the level of mtDNA damage in both tested mtDNA regions: NADH dehydrogenase subunit 1 (ND1) and NADH dehydrogenase subunit 5 (ND5). In conclusion, the results of the in vitro study revealed that T-2 toxin shows adverse effects on Hs68 cell mitochondria. T-2 toxin induces mitochondrial dysfunction and mtDNA damage, which may cause the disruption of adenosine triphosphate (ATP) synthesis and, in consequence, cell death.

## 1. Introduction

Mitochondria are essential organelles playing a key role in various cell processes. They are frequently called the “powerhouses of the cell”. This is mainly due to the fact that their primary and most well-described role is in adenosine triphosphate (ATP) production via oxidative phosphorylation (OXPHOS), which is executed by the mitochondrial electron transport chain (ETC) [1,2]. However, mitochondria are also responsible for iron–sulphur cluster formation, calcium handling, lipid metabolism, reactive oxygen species (ROS) generation, cell signaling, apoptotic activation, and mediation of cell growth and death [3,4]. The mitochondrial membrane potential (MMP) generated by proton pumps (complexes I, III, and IV) is a key indicator of mitochondrial activity. It reflects the process of electron transport and oxidative phosphorylation and provides the driving force for ATP synthesis in mitochondria [5]. Furthermore, MMP is required for mitochondrial protein import and for regulating metabolite transport [6]. A loss of MMP is a signal of bioenergetic stress and may cause the release of pro-apoptotic factors leading to cell death [7].

Mitochondria contain their own genome: mitochondrial DNA (mtDNA), housed in the mitochondrial matrix [8]. The mitochondrial genome is circular, double-stranded, and contains very few introns, and it is built of 16,569 base pairs. Mitochondrial DNA possesses a small number of genes, which encode 13 proteins, 22 transfer RNAs (tRNAs), and 2 ribosomal RNAs (rRNAs): 12S and 16S in humans. These proteins are important components of the mitochondrial respiratory chain (MRC) and of the ATP synthase complex, while tRNA and rRNA are required for translation [9]. One mitochondrion can contain 2–10 copies of mtDNA, and up to 1000 mitochondria are present per cell [10]. Transcription, translation, and replication of mtDNA, including the synthesis of ribosomal proteins, are under nuclear regulation. As a consequence, mitochondrial function depends on coordinated interaction between nuclear DNA (nDNA) and mtDNA-encoded proteins, protein assembly factors, and chaperone proteins, which are involved in protein folding and scaffolding and structural support [11]. mtDNA is inherited exclusively maternally, while the sperm-borne mitochondria are mostly degraded by autophagy after fertilization [12].

T-2 toxin belongs to the group of type A trichothecene mycotoxins and is produced by various *Fusarium* species, such as *F*. *sporotrichioides*, *F*. *poae*, and *F*. *tricinctum* [13]. T-2 mycotoxin contaminates a variety of grains, including wheat, barley, maize, rice, oat, and soybeans, and therefore poses a risk to human and animal health [14]. The European Food Safety Authority (EFSA) established new standards for T-2 toxin: a tolerable daily intake (TDI) of 0.02 μg/kg body weight (bw) per day and an acute reference dose (ARfD) of 0.3 μg/kg bw [15]. T-2 is considered one of the most toxic trichothecenes [16]. It is mainly harmful to human and animal digestive, reproductive, nervous, and immune systems. T-2 shows dermal toxicity, hematotoxicity, carcinogenic, and mutagenic effects [17]. T-2 toxin is a low molecular chemical compound with a weight of approximately 466.51 Da [18]. According to IUPAC nomenclature: 1R,9R,10R,11S,12R(-11-acetyloxy-2-(acetyloxymethyl)-10-hydroxy-1,5-dimethylspiro(8-oxatricyclo(7.2.1.02,7))dodec-5-ene-12,2′-oxirane)-4-yl)-3-methylbutanoate. The epoxy rings at the C-12 and -13 positions are responsible for toxicological activity [19]. This structure is responsible for T-2 toxin’s lipophilic character and the fact that it can be immediately absorbed by exposed cells [20]. The main effect of T-2 toxin is protein synthesis inhibition, which leads to the secondary disruption of DNA and RNA synthesis. Toxin also causes lipid peroxidation and reactive oxygen species (ROS) generation, as well as apoptosis and necrosis [21,22].

Studies have shown that T-2 toxin causes DNA damage [23,24,25]. Cells are able to use many mechanisms in order to repair their DNA; thus, the integrity of nuclear (nDNA) and mitochondrial DNA (mtDNA) can be maintained. In the absence of damage recognition or repair fails, an accumulation of DNA lesions appears [26]. mtDNA is associated with a sustained 10-fold higher level of damage in comparison to nDNA due to the lack of protective histone proteins in mitochondria [27]. The apparent lack of mtDNA repair mechanisms and also the low fidelity of the mtDNA polymerase lead to a higher mutation rate in the mtDNA compared to the nuclear genome [28]. Moreover, mtDNA is particularly susceptible to mutation due to the production of ROS in mitochondria during respiration, which can cause oxidative lesions in mtDNA [29,30]. mtDNA is more susceptible to damage than nDNA, mainly due to the fact that the entire mitochondrial genome codes expressed genes, whereas the nuclear genome contains a large amount of nontranscribed sequences. Furthermore, in contrast to nDNA, mtDNA is continuously replicated, even in terminally differentiated cells such as cardiomyocytes [31].

In our previous study performed on the human skin fibroblast Hs68 cell line, it was demonstrated that T-2 toxin in a dose and time-dependent manner had cytotoxic effect. The bioluminometry results showed that the relative levels of ATP in the treated cells decreased. Further analysis of the toxin’s impact on the apoptosis induction and necrosis processes clearly indicates the necrosis process in treated cells. This paper contains the results of an in vitro human skin fibroblast model study and reveals for the first time that T-2 toxin induces necrosis as an effect of toxicity [32].

Due to the lack of information on the T-2 molecular mechanisms of action on the skin and the effects of this toxin on mitochondria, in vitro studies with a cell line of normal human fibroblasts Hs68 were conducted in order to explain the effect of this toxin on mitochondria and mtDNA.

## 2. Results

### 2.1. Assessment of Mitochondrial Membrane Potential (MMP)

**Figure 1:**
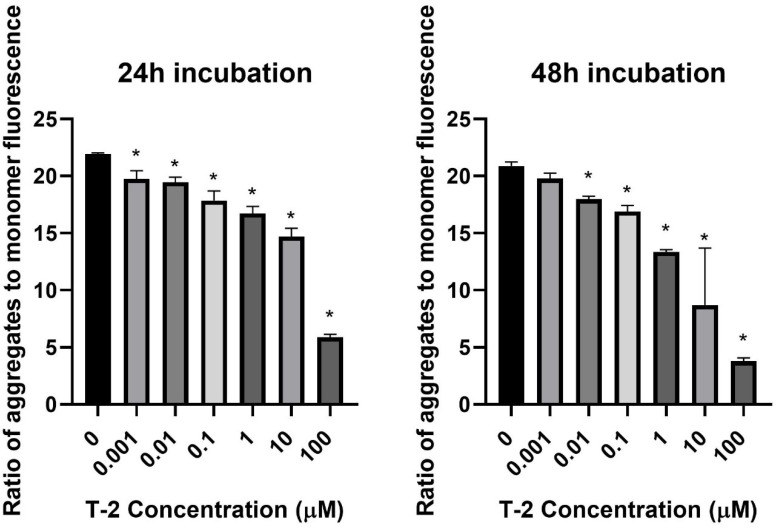
The T-2 toxin effect on the mitochondrial membrane potential of Hs68 cells estimated by the fluorescence dye JC-1 method. The values are presented as means ± SD (*n* = 6). * *p* < 0.5.

In the first step of our study, T-2 toxin’s effect on cell mitochondria’s physiological status was determined. The effect of T-2 toxin on a cell line of normal human fibroblasts Hs68 mitochondrial membrane potential was examined. MMP plays a key role in mitochondrial homeostasis through the selective elimination of dysfunctional mitochondria. The treatment of cell line resulted in a dose- and time-dependent changes of tetraethylbenzimidazolylcarbocyanine iodide (JC-1) monomer fluorescence aggregates ratio (Figure 1), which indicated a decrease in the membrane potential in cells treated with T-2 toxin.

### 2.2. Assessment of Intracellular Reactive Oxygen Species (ROS) Level

**Figure 2:**
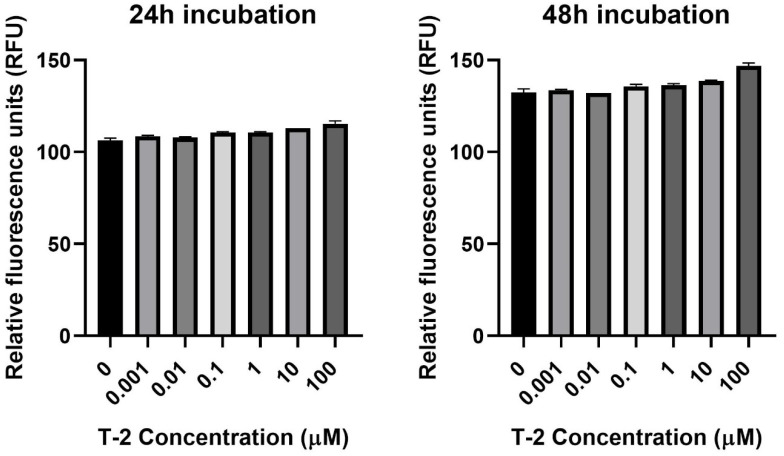
The T-2 toxin’s effect on intracellular ROS production in the Hs68 cell line, measured as the DCF fluorescence intensity. The values are presented as means ± SD (*n* = 6).

The next step of the study was the assessment of the impact of T-2 on Hs68 mitochondria by an evaluation of the intracellular generation of reactive oxygen species. In order to perform this analysis, the experiments with 2′,7′-dichlorofluorescein diacetate (DCFH-DA) dye, which oxidizes to highly fluorescent 2′,7′-dichlorofluorescein (DCF) in the presence of intracellular ROS, were executed. The incubation of Hs68 cells with T-2 toxin at all tested concentrations showed no impact on the intracellular ROS (Figure 2).

### 2.3. Mitochondrial DNA (mtDNA) Copy Number Quantification

**Figure 3:**
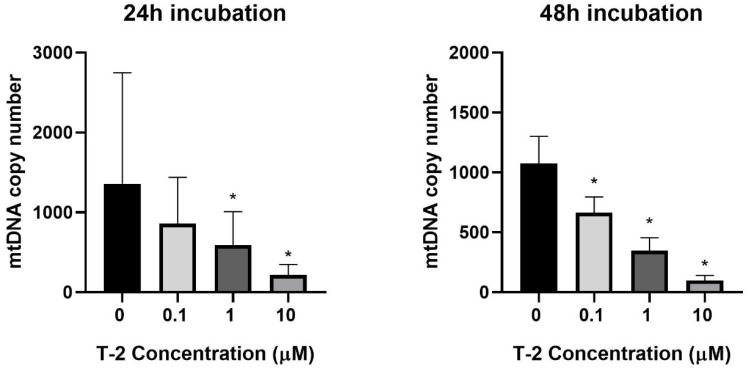
The T-2 toxin’s effect on the mitochondrial DNA copy number in the Hs68 cell line, measured by real-time quantitative polymerase chain reaction (rt-qPCR). The values are presented as means ± SD (*n* = 6). * *p* < 0.05.

The next experiments focused on the impact of T-2 toxin on the mitochondrial genome. The potential genotoxicity of T-2 was observed as a change in the mtDNA copy number. According to the cytotoxicity results, three T-2 toxin concentrations were tested: 0.1, 1, and 10 µM. The incubation of the Hs68 cells with T-2 toxin in a dose- and time-dependent manner decreased the number of mtDNA copies in the cells (Figure 3). In the case of the highest tested concentration (10 μM) and a 48 h incubation period, the level decreased by more than 100 times.

### 2.4. Determination of Mitochondrial DNA Damage

**Figure 4:**
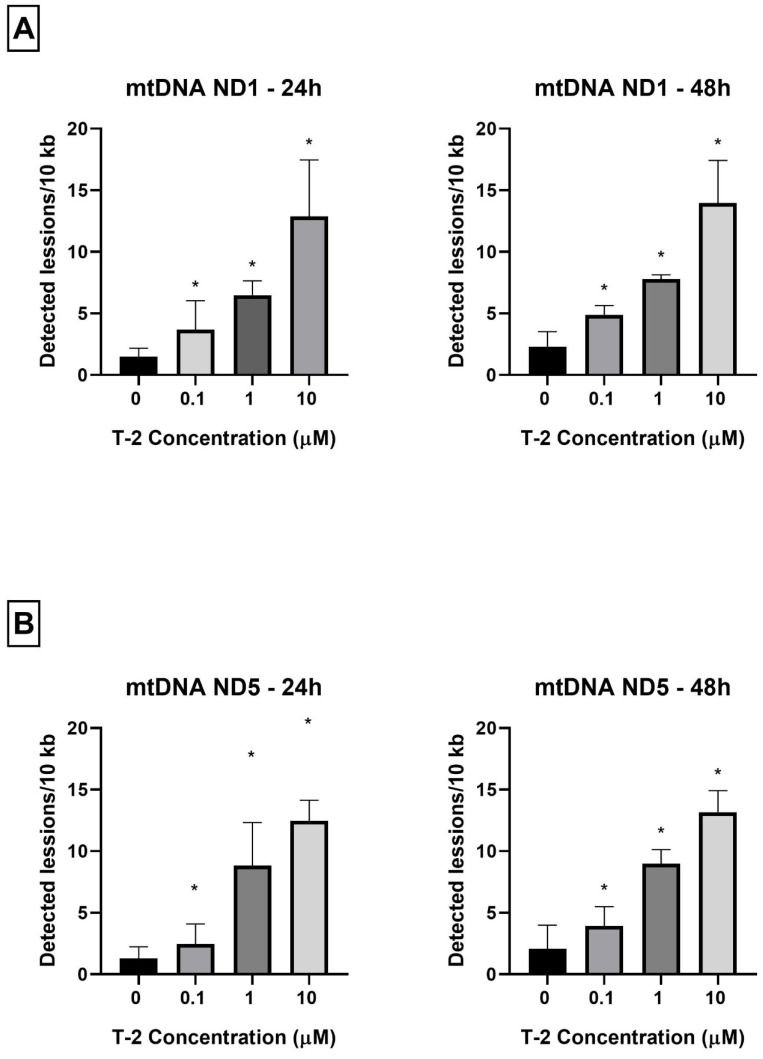
(**A**,**B**) The effect of T-2 toxin on mtDNA lesion frequency per 10 kb DNA in the ND1 and ND5 genes, estimated using SLR-qRT-PCR amplification of the total DNA from the Hs68 cells. The values are presented as means ± SD (*n* = 6). * *p* < 0.05.

As a complementary study on the determination of T-2 toxin on the mitochondrial genome of Hs68 cells, toxin genotoxicity by measurement of the mtDNA damage was performed. The mtDNA damage, by semi-long-run quantitative real-time polymerase chain reaction (SLR-qRT-PCR) amplification of the DNA isolated from cells exposed to T-2 toxin at concentrations of 0.1, 1, and 10 µM for both 24 and 48 h, was examined. The incubation of Hs68 cells with T-2 toxin in a dose- and time-dependent manner increased the level of mtDNA damage in both tested mtDNA regions—NADH dehydrogenase subunit 1 (ND1) (Figure 4A) and NADH dehydrogenase subunit 5 (ND5) (Figure 4B).

## 3. Discussion

T-2 toxin has a unique character, which was the main reason behind the initiation of our studies focused on the determination of the molecular mechanism of action of T-2 toxin in in vitro human foreskin fibroblast cell line Hs68. The first paper [32] in the series of our studies showed the necrotic potential of T-2 toxin alongside a strong reduction in ATP production by cells. Consecutively, the aim of this paper was to present the impact of T-2 toxin on mitochondrial physiology and disruption of mitochondrial DNA.

The mitochondrial membrane potential is a driving force behind the transport of ions and proteins, which is crucial for healthy mitochondrial functioning [5]. The decrease in MMP might be an indicator of cell death and a cause of various pathologies [33]. A decrease in MMP was observed in both necrotic and apoptotic cell deaths. One theory proposes that the ATP level (ATP may be the switch) determines whether cell destruction occurs by necrosis or apoptosis. In this context, necrotic cell death is solely dependent on oxidative phosphorylation for ATP. When the decrease in MMP occurs without ATP depletion, apoptosis can develop. When the decrease in MMP is related to mitochondrial disfunction and ATP depletion, necrotic cell death might occur [34]. This theory is decisively confirmed by our studies. In this study, a strong decrease in the mitochondrial membrane potential in cells treated with T-2 toxin (Figure 1) was demonstrated. In our previous paper [32], using the bioluminometry method, we observed that the treatment of cell line resulted in a dose- and time-dependent decrease in the level of luminescence, which directly corresponds to the ATP level in the sample (see Figure 3 in [32]). The samples were from the same batch of culture used in the current research. In this context, the potential mechanism of necrotic cell death caused by T-2 toxin is dependent on the mitochondrial damage caused by the ATP disturbance in oxidative phosphorylation.

ATP oxidative phosphorylation in mitochondria points to the fact that these organelles are the main cellular consumers of oxygen. The redox enzymes present in mitochondria cause the transfer of single electrons to oxygen and nonenzymatic production of O_2_^−•^. Mitochondrial damage and physiological imbalance can cause a disproportion between ROS production and removal, resulting in net ROS production [35]. The results presented in Figure 2 demonstrate that the T-2 toxin at all tested concentrations and times did not change the intracellular ROS level. This observation also confirms the fact that T-2 toxin inhibits ATP oxidative phosphorylation. In a study of the role of mitochondria in T-2-induced apoptosis of human chondrocytes, a decrease in the MMP of chondrocytes following T-2 toxin administration was shown. In addition, the ROS levels, as a mitochondrial apoptotic factor, significantly increased. In addition, caspase-3 and caspase-9 were activated in those chondrocytes [36]. This study shows that T-2 toxin can also decrease the MMP of fibroblast cells, however, with no effect on the ROS level. Our previous study on T-2 toxin’s impact on the induction of apoptosis and necrosis processes in a fibroblast cell line showed a lack of caspase-3, which controls the fragmentation of DNA and morphological changes of apoptosis, and caspase-7, which is related to the loss of cellular viability [32]. The lack of changes in the ROS level and the activity of caspase-3/7, which are involved in the apoptosis process, indicate a necrosis process as the impact of T-2 toxin on the fibroblast cells. Additionally, experiments on the same samples using the double-staining flow cytometry method, which were presented in our previous paper [32], demonstrated that the T-2 mycotoxin resulted in both a dose- and time-dependent increase in propidium iodide (PI) fluorescence in cells. At the highest tested concentration of the toxin, 100 µM, the % of PI-stained cells was 79% after 24 h of incubation and 93% after 48 h of incubation.

The next steps of the research presented in this paper focused on molecular damage, which can be caused by the presence of T-2 toxin in mitochondria. mtDNA is crucial for mitochondrial physiological status and function. mtDNA replication is independent from the cell cycle and from nuclear DNA replication. This process is conducted by the encoded polymerase γ, the only DNA polymerase found in mitochondria [37]. A sufficient number of mtDNA copies is necessary to meet their specific requirements for the generation of cellular energy through oxidative phosphorylation [38]. mtDNA is susceptible to reactive species generated from cellular metabolism and to environmental agents, including therapeutic drugs, radiation, and industrial byproducts [39]. The significance of mtDNA in homeostasis is evidenced by the fact that many diseases are caused by mtDNA depletion or mutations [40]. Mitochondrial dysfunctions are associated with numerous human diseases, such as neurodegenerative disorders, neurometabolic diseases, cardiovascular disorders, cancer, or obesity [41]. This study shows that T-2 toxin can cause a significant decline in the number of mtDNA copies (Figure 3). This observation confirmed previous conclusions concerning mitochondrial damage and the disturbance of ATP production induced by T-2. The molecular mechanism of this action is probably linked to mtDNA damage. As a final confirmation of the responsibility of T-2 toxin for mtDNA damage, the analysis of mtDNA lesions in two genes—ND1 and ND5—was performed. The mitochondrial ND1 gene is translated into the NADH-ubiquinone oxidoreductase chain 1 (ND1) [42], whereas the ND5 gene encodes the NADH-ubiquinone oxidoreductase chain 5 protein [43]. Both of these proteins are subunits of NADH dehydrogenase—the largest of the five complexes of the electron transport chain [44]. The results of our research presented in the current paper demonstrate that the T-2 toxin induced mtDNA damage in both of the tested mtDNA genes—ND1 (Figure 4A) and ND5 (Figure 4B). This observation confirmed the damaging effect of T-2 toxin on mitochondrial function at the molecular level, which can, consequently, lead to the inhibition of ATP production [32] and cell necrosis. 

The studies performed by Pace et al. [45] demonstrated that T-2 toxin, in dose-dependent manner, inhibited protein synthesis in isolated rat liver mitochondria. The concentration of toxin, which inhibited 50% of protein synthesis, was approximately 0.05 μM. T-2 toxin was presented as an inducer of mitochondrial dysfunction and an inhibitor of ATP synthesis in cardiomyocytes [46]. Many studies have also demonstrated that T-2 toxin induced MMP loss [36,47,48,49].

It has been shown that other fungal toxins can also cause mitochondrial and mtDNA damage. A study on ducklings showed that the administration of aflatoxin B_1_ (AFB_1_) can induce hepatic mitochondrial antioxidant dysfunction. An analysis of the ducklings’ liver tissue revealed morphological changes, including fat necrosis, steatosis, and the formation of lymphoid nodules, with infiltrated lymphocytes. AFB_1_ exposure induced mitochondrial swelling with increased opening of the liver mitochondrial permeability transition pore. In addition, a sequence analysis of the mtDNA D-loop region indicated that AFB1 induces mtDNA damage. Mutations in the D-loop region interfere with a transcription of the entire mtDNA genome and possibly cause potent alterations in mitochondrial function [50].

In this paper, for the first time, we have demonstrated the molecular mechanism of mitochondrial disfunction induced by T-2 toxin. The damaging of mitochondrial DNA can be responsible for ATP synthesis disruption and can lead to cell death.

## 4. Materials and Methods

### 4.1. Reagents

Dimethyl sulfoxide (DMSO) and T-2 toxin from Fusarium sp. (cat. No T4887) were obtained from Sigma-Aldrich Chemical Co. (St. Louis, MO, USA). Dulbecco’s modified Eagle medium (DMEM) with 4.5 g/L glucose and L-glutamine, heat-inactivated fetal bovine serum (FBS), penicillin–streptomycin mixture, and PBS (1X) without calcium or magnesium were purchased at Lonza (Basel, Switzerland). The 5,5′,6,6′-tetrachloro-1,1′,3,3′-tetraethylbenzimidazol-carbocyanine iodide (JC-1) probe, Hank’s Balanced Salt Solution (HBSS), 2′,7′-dichlorodihydrofluorescein diacetate (H_2_DCFDA) probe and JC-1 dye were from Thermo Fisher Scientific (Waltham, MA, USA). All other chemicals were of molecular grade or the highest quality available.

### 4.2. Cell Culture

In this study, the human foreskin fibroblast cell line Hs68 (ATCC^®^ CRL-1635™), obtained from the American Type Culture Collection (ATCC™, Manassas, VA, USA), was used. The Hs68 cells were cultured in DMEM supplemented with 100 units of potassium penicillin and 100 μg of streptomycin sulphate per 1  mL of culture media, 10% (*v*/*v*) FBS and kept in an incubator with a 5% CO_2_ atmosphere at 100% humidity and 37 °C. 

### 4.3. Assessment of Mitochondrial Membrane Potential (MMP)

The MMP was assessed using tetraethylbenzimidazolylcarbocyanine iodide (JC-1) cationic carbocyanine dye, which accumulates in the mitochondrial membrane in a potential-dependent manner. A high potential of the inner mitochondrial membrane leads to the formation of dye aggregates. Depolarization and permeation of the mitochondrial membrane lead to a breakdown of aggregates into monomers emitting green fluorescence with excitation and emission values of 485 and 538 nm, respectively [33]. The cells used for analysis were seeded into 96-well, black plates with transparent bottoms (Greiner Bio-One, Kremsmünster, Austria) at a density of 1 × 10^5^ cells/well in 50 μL culture medium and allowed to adhere for 12 h. Next, cells were incubated with T-2 toxin in a concentration range of 0.001 to 100 µM for 24 and 48 h. The untreated cells were used as a control. After treatment, cells were preincubated for 30 min with 5 μM JC-1 dye in HBBS in a 5% CO_2_ atmosphere at 37 °C. The cells were centrifuged (300× *g* for 10 min at 22 °C) and washed twice with the HBSS. The fluorescence was measured on a Bio-Tek Synergy HT Microplate Reader (Bio-Tek Instruments, Winooski, VT, USA), with filter pairs of 530/590 and 485/538 nm. The results are presented as a ratio of the aggregates to monomer fluorescence.

### 4.4. Assessment of Intracellular ROS Level

The relative level of intracellular ROS in cells was measured using the 2′,7′-dichlorodihydrofluorescein diacetate (H2DCFDA) dye. The nonpolar, cell-permeable H2DCFDA was diffused into cells and deacetylated by cellular esterase to the polar and membrane impermeable form 2′,7′-dichlorodihydrofluorescein (H2DCF). H2DCF is nonfluorescent, but in the presence of intracellular ROS, it rapidly oxidizes to highly fluorescent 2′,7′-dichlorofluorescein (DCF). The fluorescence intensity is proportional to the ROS levels within the cell cytosol [51]. The cells used for analysis were seeded into 96-well plates at 1 × 10^5^ cells/well in 50 μL culture medium and cultured at 37 °C for 12 h in a 5% CO_2_-containing environment. Next, the cells were treated with T-2 toxin in the concentration range 0.001 to 100 µM and incubated for 24 and 48 h. The untreated cells were used as the control. After treatment, the cells were incubated with 5 µM of 2′,7′-dichlorofluorescein diacetate (DCFH-DA) (prepared in Tyrode’s Ca2+/Mg2+ free buffer) at 37 °C for 45 min. The fluorescence was measured at a 480 nm excitation wavelength and a 510 nm emission wavelength, using a Bio-Tek Synergy HT Microplate Reader (Bio-Tek Instruments, Winooski, VT, USA), and expressed as the intensity of the DCF fluorescence.

### 4.5. Isolation of Total Genomic DNA from Cell Lines

To perform the analysis, cells were seeded at 3 × 10^6^ cells per well and left in the incubator for 12 h before the treatment procedures. Then, they were incubated with T-2 toxin (Sigma-Aldrich Chemical Co., St. Louis, MO, USA) in the concentration range 0.1 to 10 µM for 24 and 48 h. The working solutions of the T-2 toxin were made by the direct dilution of the toxin in culture medium. The untreated cells were used as the control. The total genomic DNA (mitochondrial and nuclear) from the cell pellets was isolated using the commercially available EXTRACT ME RNA & DNA KIT (BLIRT S.A., Gdansk, Poland), according to the producer’s protocol. The DNA concentrations were determined by spectrophotometric measurement of the absorbance at 260 nm. The purities were calculated by a A260/A280 ratio using the Bio-Tek Synergy HT Microplate Reader (Bio-Tek Instruments, Winooski, VT, USA). The purified DNA was stored at −30 °C until further analysis.

### 4.6. Mitochondrial DNA Copy Number Quantification

The relative number of copies of human mtDNA using nDNA content as a standard was evaluated by quantitative real-time PCR (qRT-PCR). For quantification, two primer pairs for mtDNA detection (*ND1* and *ND5*) and two primer pairs for nDNA detection (SERPINA1 and SLCO2B1) were selected. All primers were designed with the Primer3 software (http://bioinfo.ut.ee/primer3-0.4.0/ (accessed on 15 July 2021)) and synthesized by Sigma-Aldrich Chemical Co. (St. Louis, MO, USA). Complete nucleotide sequences for each gene were obtained from the ENSEMBL database (https://ensembl.org/ (accessed on 15 July 2021)). The mitochondrial *ND1* (124 bp fragment size) and *ND5* genes (124 bp fragment size) were amplified using the pairs of primers (forward primer 5′-CCTAAAACCCGCCACATCTA-3′ and reverse primer 5′-GCCTAGGTTGAGGTTGACCA-3′; forward primer 5′-AGGCGCTATCACCACTCTGT-3′ and reverse primer 5′-TTGGTTGATGCCGATTGTAA-3′, respectively). The amount of nuclear DNA was determined using the SLCO2B1 (135 bp fragment size) and SERPINA1 (148 bp fragment size) genes as a reference: forward primer 5′-GATCCCAGCCAGTGGACTTA-3′, reverse primer: 5′-CCTGAAGCTGAGGAGACAGG-3′; forward primer 5′-TGCAGCTTCCTCTTCACAGA-3′ and reverse primer 5′-CTCAGCCCCAAGTATCTCCA-3′, respectively.

The qRT-PCR was performed using a CFX96 Real-Time PCR Detection System (Bio-Rad Laboratories, Hercules, CA, USA). The qRT-PCR reaction mix was performed in a total volume of 10 µL with 1 × Power SYBR Green PCR Master Mix (Thermo Fisher Scientific, Waltham, MA, USA), 250 nM of each primer, and 1 μL (5 ng) of genomic DNA. The cycling sequence was as follows: enzyme activation at 95 °C for 10 min, then 40 cycles of 3 s denaturation at 95 °C, 30 s annealing at 65 °C, and 15 s extension at 72 °C, with plate reading at this step. The reactions were performed in duplicate and included a negative control (without template DNA). The cycle threshold (Ct) values were computed automatically and then analyzed using CFX Manager^TM^ Software, version 3.1 (Bio-Rad Laboratories, Hercules, CA, USA). The relative nDNA copy number was calculated using the formula 2^ΔCt1^ and ^ΔCt2^, where ΔCt1 = Ct for SLCO2B1 − Ct for ND1; ΔCt2 = Ct for SERPINA1 − Ct for ND5. The relative mtDNA copy number was calculated using the formula 2^ΔCt1^ and ^ΔCt2^, where ΔCt1 = Ct for SLCO2B1 − Ct for ND1; ΔCt2 = Ct for SERPINA1 − Ct for ND5.

### 4.7. Determination of Mitochondrial DNA Damage

The assessment of the mtDNA damage was performed using a semi-long-run quantitative RT-PCR (SLR-qRT-PCR) [52]. The levels of DNA lesions in the tested region of the mitochondrial genome were measured using two fragments of different lengths (i.e., short and long fragments) located in the same mitochondrial genomic region. The sequences of all primers used in this study are listed in Table 1. All primers were designed using Primer3 software (http://bioinfo.ut.ee/primer3–0.4.0/ (accessed on 17 July 2021)) and synthesized by Sigma-Aldrich (St. Louis, MO, USA). The complete nucleotide sequences for each gene were taken from the ENSEMBL database (https://ensembl.org/ (accessed on 17 July 2021)).

The SLR-qRT-PCR amplification was performed using a CFX96 Real-Time PCR Detection System (Bio-Rad Laboratories, Hercules, CA, USA). The SLR-qRT-PCR reaction mix was performed in a total volume of 10 µL with 1 × Power SYBR Green PCR Master Mix (Thermo Fisher Scientific, Waltham, MA, USA), 250 nM of each primer, and 5 ng of template DNA. The PCR reaction conditions were enzyme activation at 95 °C for 10 min followed by up to 40 cycles of 15 s denaturation at 95 °C, 30 s annealing at 65 °C, and 15 s extension at 72 °C (for short amplicons) or 45 s at 72 °C (for long amplicons). The Ct values were computed automatically and then analyzed using CFX Manager^TM^ software (version 3.1). The DNA damage was calculated as lesion per 10 kb DNA of each region, by including the size of a particularly long fragment. The following formula was used: lesion per 10 kb DNA = (1 − 2^−(Δlong − Δshort)^) × 10,000 (bp)/size of long fragment (bp), where ∆long and ∆short show differences in the Ct value between treated samples and nontreated cells (control). The DNA isolated from the controls was used as a reference, while the Ct of the large and small mitochondrial fragments was used for the DNA damage quantification.

### 4.8. Data Analysis

All obtained experimental values were elaborated using Microsoft Excel software (Redmond, WA, USA) and stated as mean values ± standard deviations (SDs). The statistical analysis was performed using StatsDirect statistical software V. 2.7.2. (Cheshire, UK). All results were analyzed according to the normality of the distribution using the Shapiro–Wilk test. The results were examined according to equality of variance via Levene’s test. The significance of the differences among the values was analyzed using ANOVA, Tukey’s range test (for data with normal distribution and equality of variance), or the Kruskal–Wallis test; *p* < 0.05 was accepted as statistically significant [53,54].

## 5. Conclusions

We demonstrated for the first time that in an in vitro human skin fibroblast model, T-2 toxin has an influence on the physiological role of the fibroblast mitochondrial Hs68 cell line. Based on our current and previous observations, we conclude that T-2 toxin reduces the mitochondrial membrane potential and inhibits the production of ATP. The T-2 toxin-induced damage to mitochondrial functions is a probable major cause of necrotic death of Hs68 cells. The molecular mechanism of this toxic effect is related to mtDNA damage, leading to the maintenance of its integrity, which is critical for proper organellar function.

## Figures and Tables

**Table 1:** Specifications of the SLR-qRT-PCR primers used for the quantification of mitochondrial DNA damage.

Target Gene Symbol	Forward Primer Sequences (5′→3′)	Reverse Primer Sequence (5′→3′)	Amplicon Length (bp)
*ND1* (mitochondrially encoded NADH: ubiquinone oxidoreductase core subunit 1)	Long fragment: ATGGCCAACCTCCTACTCCT	Long fragment: GATGAGTGTGCCTGCAAAGA	1214
	Small fragment: CCTAAAACCCGCCACATCTA	Small fragment: GCCTAGGTTGAGGTTGACCA	124
*ND5* (mitochondrially encoded NADH: ubiquinone oxidoreductase core subunit 5)	Long fragment: TCCAACTCATGAGACCCACA	Long fragment: AGGTGATGATGGAGGTGGAG	1156
	Small fragment: AGGCGCTATCACCACTCTGT	Small fragment: TTGGTTGATGCCGATTGTAA	124

## Data Availability

Not applicable.
